# Is the Number of Missing Teeth Associated With Mortality? A Longitudinal Study Using a National Health Screening Cohort

**DOI:** 10.3389/fmed.2022.837743

**Published:** 2022-06-21

**Authors:** So Young Kim, Chang Ho Lee, Dae Myoung Yoo, Mi Jung Kwon, Ji Hee Kim, Joo-Hee Kim, Soo-Hwan Byun, Bumjung Park, Hyo-Jeong Lee, Hyo Geun Choi

**Affiliations:** ^1^Department of Otorhinolaryngology-Head and Neck Surgery, CHA Bundang Medical Center, CHA University, Seongnam, South Korea; ^2^Hallym Data Science Laboratory, Hallym University College of Medicine, Anyang, South Korea; ^3^Department of Pathology, Hallym University College of Medicine, Anyang, South Korea; ^4^Department of Neurosurgery, Hallym University College of Medicine, Anyang, South Korea; ^5^Division of Pulmonary, Allergy and Critical Care Medicine, Department of Medicine, Hallym University College of Medicine, Anyang, South Korea; ^6^Department of Oral and Maxillofacial Surgery, Dentistry, Hallym University College of Medicine, Anyang, South Korea; ^7^Research Center of Clinical Dentistry, Hallym University Clinical Dentistry Graduate School, Chuncheon, South Korea; ^8^Department of Otorhinolaryngology-Head and Neck Surgery, Hallym University College of Medicine, Anyang, South Korea

**Keywords:** tooth loss, mortality, risk factors, cohort studies, epidemiology

## Abstract

This study aimed to estimate the risk of mortality related to the number of missing teeth in a South Korean population. The ≥ 40-year-old population of the Korean National Health Insurance Service-Health Screening Cohort 2002–2003 was analyzed. Participants were selected from a total of 220,189 participants and included in groups of 0 teeth lost, 1–2 teeth lost, and ≥ 3 teeth lost. Among the total population, 17,211 participants were included in no missing teeth, 1–2 missing teeth, and ≥ 3 missing teeth and were randomly matched 1:1:1 for age and sex. Mortality from specific causes and all-cause mortality were compared among the groups. The hazard ratio (HR) of the number of missing teeth for all-cause mortality or each cause of mortality was analyzed using Cox proportional hazard models. According to the cause of death, the HRs for metabolic disease, digestive disease, and trauma were greater in the group with ≥ 3 missing teeth than in the no missing teeth group. The group with ≥ 3 missing teeth indicated a 1.19-fold higher HR for all-cause mortality than the no missing teeth group [95% confidence intervals (95% CIs) = 1.12–1.27, *P* < 0.001]. The group with 1- 2 missing teeth did not show a higher HR for all-cause mortality. In the group with 1–2 missing teeth, the HRs for mortality from mental disease and digestive disease were higher than those in the no missing teeth group. The group with 1–2 missing teeth did not show a higher HR for all-cause mortality. The number of missing teeth was linked with a higher risk of mortality. For specific causes of mortality, mortality from metabolic disease, digestive disease, and trauma was higher in the participants with the number of missing teeth.

## Introduction

The prevalence of edentulism was estimated at 2.4% in the general population (1). The prevalence of tooth loss is increasing with aging (2). Tooth loss is related to many health-related problems (3). The physiological roles of teeth, such as mastication, speaking, and facial expressions, can be impaired when teeth are lost. Due to dysfunctions in mastication and conversations, nutritional intake and social activities can be hindered. In addition, a meta-analysis estimated the unfavorable quality of life in individuals with tooth loss, even in studies with participants with 21–24 teeth (3). These health problems and poor quality of life could increase morbidities in patients with tooth loss.

The number of missing teeth may influence the risk of morbidities in elderly individuals. Metabolic disease and digestive disease can be associated with the number of missing teeth due to impaired mastication and nutritional intake in participants with missing teeth. Having <20 teeth was connected with a greater risk of incidental falls (OR = 2.50, 95% CI = 1.21–5.17) and functional disability (OR = 1.21, 95% CI = 1.06–1.40) in elderly individuals (4, 5). Although there is no research on the association of the number of missing teeth with trauma, the increased risk of incidental falls and functional disability could increase the risk of trauma in participants with the number of missing teeth. In addition, having <20 teeth and masticatory dysfunction were connected with a greater risk of cognitive deficits in a meta-analysis (OR = 2.24, 95% 95% CI = 1.73–2.90) (6). Another meta-analysis demonstrated a 1.48 times greater risk of cognitive impairment (95% CI = 1.18–1.87) in individuals with missing teeth with a dose-response association according to the number of missing teeth from 4 to 32 teeth or 0 to 32 teeth (7, 8). A prospective cohort study estimated a 1.85 times greater risk of dementia in Japanese elderly individuals with few teeth (95% CI = 1.04–3.31) (9). Thus, it can be presumed that missing teeth could impair many aspects of daily life and impact mortality. Indeed, several researchers have suggested an increased mortality rate in individuals with missing teeth (10–12). However, most previous studies have focused on all-cause or cardiovascular and/or cancer-related mortality and have not considered various causes of mortality that are possibly affected by missing teeth. In addition, although many previous studies have examined the effects of a considerable number of missing teeth (<20 teeth remaining or edentation) on mortality, (1, 4, 5, 10–12) only a few prior studies have suggested the effects of a small number of missing teeth on morbidities (7, 8).

We hypothesized that the number of missing teeth, even if it is as small as 1–2, might be associated with a higher risk of mortality. The association of mortality with the number of missing teeth was hypothesized to be different according to the cause of death. To test these hypotheses, the mortality rates were analyzed according to the presence of the number of missing teeth. For secondary outcomes, the association of the number of missing teeth with mortality rates was analyzed according to the cause of death.

## Methods

### Ethics

The Ethics Committee of Hallym University (2019-10-023) permitted the current study. Written informed consent was not required by the Institutional Review Board. All analyses conformed to the guidelines of the Ethics Committee of Hallym University.

### Study Population and Participant Selection

**Figure 1:**
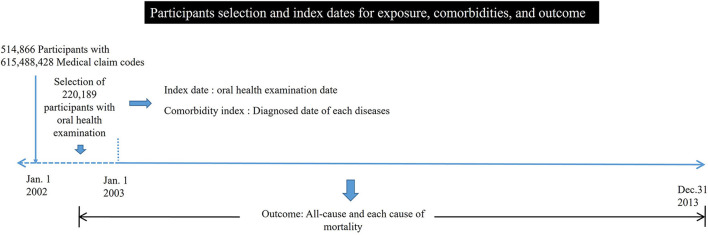
The participant selection and index date of exposure in the current study.

**Figure 2:**
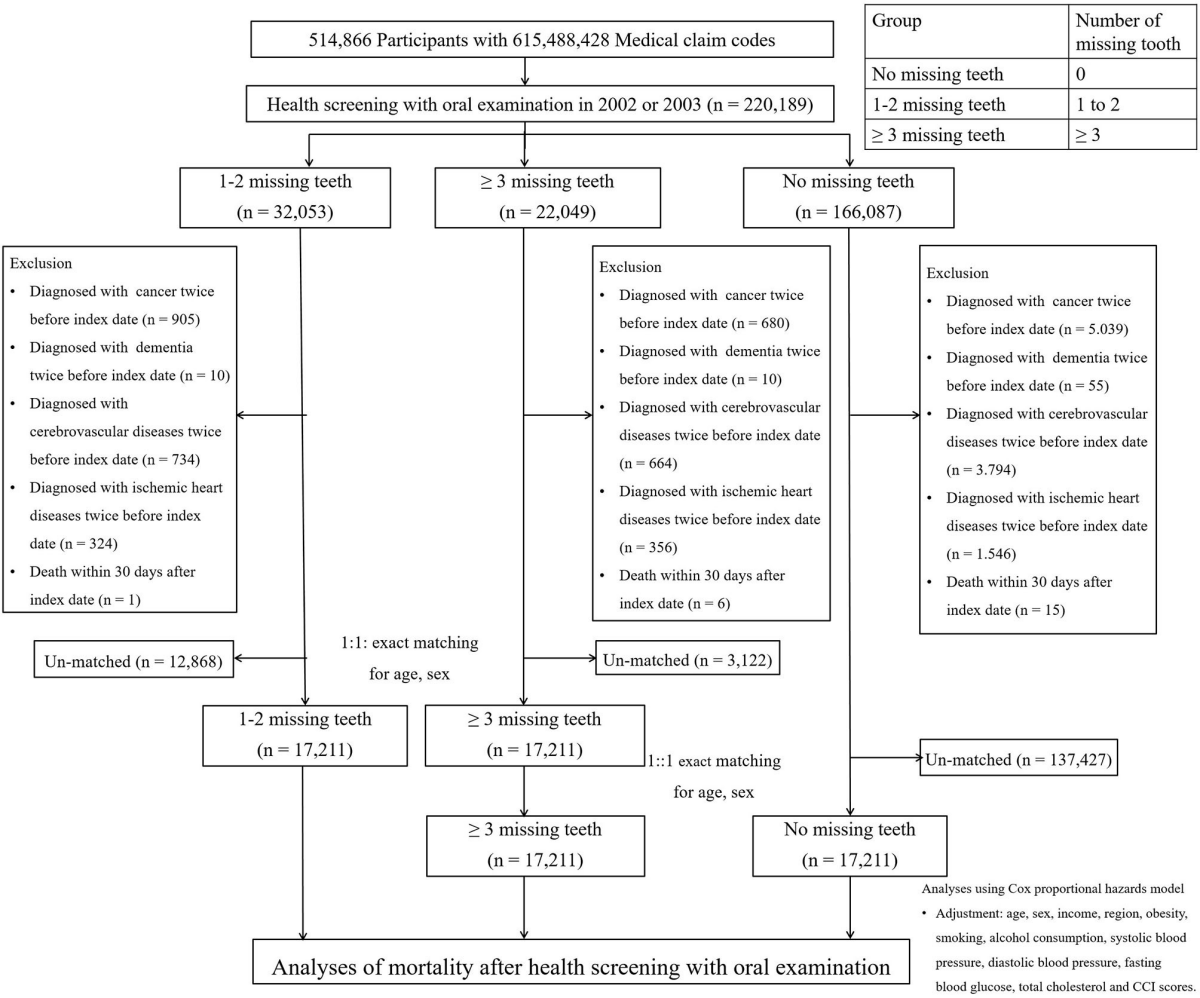
Design of the current study. A total of 17,211 participants included in the groups of ≥ 3 missing teeth, 1–2 missing teeth, and no missing teeth were matched 1:1:1 for age and sex.

The Korean National Health Insurance Service-Health Screening Cohort data were analyzed (13). Among a total of 514,866 participants with 615,488,428 medical claim codes, participants with missing teeth, including wisdom teeth, were chosen from the 220,189 participants with oral health examination data in 2002 or 2003 (group ≥ 3 missing teeth = 22,049; 1–2 missing teeth = 32,053; no missing teeth = 166,087). Participants with 2 or more morbidity diagnoses were excluded to attenuate the impacts of underlying morbidities on mortality in addition to missing teeth. In Korea, due to the national health insurance system, diagnostic codes can be initially registered before diagnostic examinations to reimburse medical costs. Thus, participants with 2 or more diagnostic codes were excluded. Participants who were diagnosed with cancer at least twice before the oral health examination date were excluded (a group with ≥ 3 missing teeth = 680; a group with 1–2 missing teeth = 905; a group with no missing teeth = 5,039). Participants who were diagnosed with dementia at least twice before the oral health examination date were excluded (a group with ≥ 3 missing teeth = 10; 1–2 missing teeth group = 10; no missing teeth group = 55). Participants who were diagnosed with cerebrovascular diseases at least twice before the oral health examination date were excluded (a group with ≥ 3 missing teeth = 664; a group with 1–2 missing teeth = 734; a group with no missing teeth = 3,794). Participants who were diagnosed with ischemic heart disease at least twice before the oral health examination date were excluded (a group with ≥ 3 missing teeth = 356; a group with 1–2 missing teeth = 324; a group with no missing teeth = 1,546). Participants who died within 30 days after the oral health examination date were excluded (a group with ≥ 3 missing teeth = 6; a group with 1–2 missing teeth = 1; a group with no missing teeth = 15) to minimize the mortality cases due to pre-existing morbidities that were not related to missing teeth. The date of the oral health examination was considered the index date (Figure 1). Participants in the group with ≥ 3 missing teeth, those in the group with 1–2 missing teeth, and those in the group with no missing teeth were 1:1:1 matched for age and sex. During the matching process, each group was randomly chosen from the total population. During the matching process, 3,122 participants in the group with ≥ 3 missing teeth, 12,868 participants in the group with 1–2 missing teeth, and 138,427 participants in the group with no missing teeth were excluded. Finally, 17, 211 participants in the group with ≥ 3 missing teeth, the group with 1–2 missing teeth, and the group with no missing teeth were matched 1:1:1 (Figure 2).

### Exposure

The number of missing teeth at baseline (index date) was ascertained by dentists during an oral health examination. Participants were classified into the “≥3 missing teeth group” for the individuals with ≥ 3 missing teeth; “1–2 missing teeth group” for the individuals with 1–2 missing teeth; and “group with no missing teeth” for the individuals with 0 missing teeth. The distribution of the number of missing teeth among the total study participants is described in Supplementary Table 1.

### Outcome

The outcomes were all-cause mortality, and each cause of mortality is from the Korean Standard Classification of Disease. The specific causes of mortality included infection, neoplasm, metabolic disease, mental disease, neurological disease, circulatory disease, respiratory disease, digestive disease, muscular disease, genitourinary disease, trauma, and other diseases. Each cause of mortality was categorized based on the ICD-10 codes. Cancer was selected as assigned codes of C00 to D49, based on the International Classification of Disease (ICD)-10 codes. Dementia was selected as assigned codes of G30 or F00, based on the ICD-10 codes. Cerebrovascular diseases were selected as assigned codes of I60 to I60, based on the ICD-10 codes. Ischemic heart diseases were selected as assigned codes of I20 to I25, based on the ICD-10 codes. The ICD-10 codes of all causes of mortality are listed in Supplementary Table 2.

The follow-up period was defined as the period from the index date to the date of mortality.

### Covariates

Age groups were classified according to 5-year intervals. Five income groups were defined using health claims data (14). Urban and rural regions of residence were defined based on the administrative divisions (15). Tobacco smoking, alcohol consumption, and obesity using BMI (body mass index, kg/m^2^) were examined and categorized as never, past, and current smokers (16). Systolic blood pressure (BP), diastolic BP, fasting blood glucose (FBG), and total cholesterol levels were examined. Missing BMI [*n* = 5 (0.010%)], systolic blood pressure [*n* = 1 (0.002%)], diastolic blood pressure [*n* = 1 (0.002%)], fasting blood glucose [*n* = 17 (0.033%)], and total cholesterol [*n* = 21 (0.041%)] levels were substituted by the average of each variable from the study population.

Comorbidities were scored using the Charlson Comorbidity Index (CCI) (17). The CCI has been widely used to measure disease burden using the following 17 comorbidities: myocardial infarction, congestive heart failure, peripheral vascular disease, cerebrovascular disease, hemiplegia or paraplegia, dementia, chronic pulmonary disease, rheumatologic disease, peptic ulcer disease, diabetes without chronic complications, diabetes with chronic complications, renal disease, any malignancy, including leukemia and lymphoma, metastatic solid tumor, mild liver disease, moderate or severe liver disease, and acquired immunodeficiency syndrome (AIDS) (17).

### Statistical Analyses

The chi-squared test for categorical variables (income level, region of residence, obesity, smoking status, alcohol consumption, and death) and ANOVA for continuous variables (systolic BP, diastolic BP, FBG, total cholesterol, and CCI score) were applied to the groups of those with ≥ 3 missing teeth, those with 1–2 missing teeth, and those with no missing teeth. The chi-squared test was applied to compare disease-specific mortality rates between groups of the number of missing teeth.

Cox proportional hazard models were applied to examine the hazard ratios (HRs) and 95% confidence intervals (CIs) for mortality in individuals with missing teeth. In this analysis, crude (simple) and adjusted (for age, sex, income, region, obesity, smoking status, alcohol consumption, systolic blood pressure, fasting blood glucose, total cholesterol, and CCI scores) models were used.

Kaplan–Meier analysis and the log-rank test were applied to examine the cumulative probability of mortality in the ≥ 3 missing teeth, 1–2 missing teeth, and no missing teeth groups.

The E-value was evaluated to assume the effects of unconsidered confounders (18–21). An E-value higher than the association of unconsidered possible confounders, with the exposure/outcome, indicated a valid relationship between tooth loss and the risk of mortality in this study.

Subgroup analyses were performed for age (< 55 years old and ≥ 55 years old), obesity (BMI of < 23 and BMI of ≥ 23), and blood pressure (SBP of < 140 mmHg vs. SBP of ≥ 140 mmHg) using the adjusted model.

Two-sided analyses were applied. The alpha level was set as 0.05, and a *P*-value less than the alpha level was considered to indicate a significant difference. Analyses were conducted with SAS version 9.4 (SAS Institute Inc., Cary, NC, USA).

## Result

The rates of age and sex were the same owing to matching, while the rates of the other variables were different (Table 1).

**Table 1:** General characteristics of participants.

**Characteristics**	**Total participants**			
	**≥3 Missing teeth**	**1–2 Missing teeth**	**No missing teeth**	***P*-value**
Age (years)				1.000
40**–**44	2,108 (12.3)	2,108 (12.3)	2,108 (12.3)	
45**–**49	2,760 (16.0)	2,760 (16.0)	2,760 (16.0)	
50**–**54	3,466 (20.1)	3,466 (20.1)	3,466 (20.1)	
55**–**59	2,848 (16.6)	2,848 (16.6)	2,848 (16.6)	
60**–**64	3,437 (20.0)	3,437 (20.0)	3,437 (20.0)	
65**–**69	1,509 (8.8)	1,509 (8.8)	1,509 (8.8)	
70**–**74	839 (4.9)	839 (4.9)	839 (4.9)	
75**–**79	217 (1.3)	217 (1.3)	217 (1.3)	
80**–**84	27 (0.2)	27 (0.2)	27 (0.2)	
Sex				1.000
Male	10,290 (59.8)	10,290 (59.8)	10,290 (59.8)	
Female	6,921 (40.2)	6,921 (40.2)	6,921 (40.2)	
Income (groups)				<0.001^*^
1 (lowest)	3,943 (22.9)	3,328 (19.3)	3,939 (22.9)	
2	3,336 (19.4)	2,893 (16.8)	3,340 (19.4)	
3	3,224 (18.7)	3,135 (18.2)	3,224 (18.7)	
4	3,322 (19.3)	3,568 (20.7)	3,322 (19.3)	
5(highest)	3,386 (19.7)	4,287 (24.9)	3,386 (19.7)	
Region of residence (groups)				<0.001^*^
Urban	5,408 (31.4)	6,831 (39.7)	5,408 (31.4)	
Rural	11,803 (68.6)	10,380 (60.3)	11,803 (68.6)	
Obesity (groups)^†^				<0.001^*^
Underweight	610 (3.5)	374 (2.2)	440 (2.6)	
Normal	6,370 (37.0)	5,651 (32.8)	6,218 (36.1)	
Overweight	4,388 (25.5)	4,848 (28.2)	4,576 (26.6)	
Obese I	5,339 (31.0)	5,786 (33.6)	5,519 (32.1)	
Obese II	504 (2.9)	552 (3.2)	458 (2.7)	
Smoking status (groups)				<0.001^*^
Never	10,553 (61.3)	11,384 (66.1)	11,583 (67.3)	
Past smoker	1,421 (8.3)	1,515 (8.8)	1,647 (9.6)	
Current smoker	5,237 (30.4)	4,312 (25.1)	3,981 (23.1)	
Alcohol (frequency)				< 0.001^*^
< 1 time a week	11,960 (69.5)	12,041 (70.0)	12,279 (71.3)	
≥ 1 time a week	5,251 (30.5)	5,170 (30.0)	4,932 (28.7)	
SBP (mmHg)	129.15 (18.78)	129.22 (18.61)	128.10 (18.07)	<0.001^*^
DBP (mmHg)	80.32 (11.60)	80.63 (11.62)	80.11 (11.43)	<0.001^*^
Fasting blood glucose (mg/dL)	101.29 (42.63)	99.16 (34.94)	97.67 (34.01)	<0.001^*^
Total cholesterol (mg/dL)	197.85 (40.12)	200.66 (38.79)	200.47 (39.20)	<0.001^*^
CCI score (group)	0.92 (1.67)	0.82 (1.58)	0.83 (1.60)	<0.001^*^
Death				<0.001^*^
No	14,924 (86.7)	15,475 (89.9)	15,330 (89.1)	
Yes	2,287 (13.3)	1,736 (10.1)	1,881 (10.9)	

*CCI, Charlson comorbidity index; Data are presented as mean (standard deviation) or numbers (percentages).*

* *Chi-squared test for categorical variable, ANOVA for continuous variable. Significance at P < 0.05.*

† *Obesity (BMI, body mass index, kg/m^2^) was categorized as < 18.5 (underweight), ≥ 18.5– < 23 (normal), ≥ 23– < 25 (overweight), ≥ 25– < 30 (obese I), and ≥ 30 (obese II)*.

**Figure 3:**
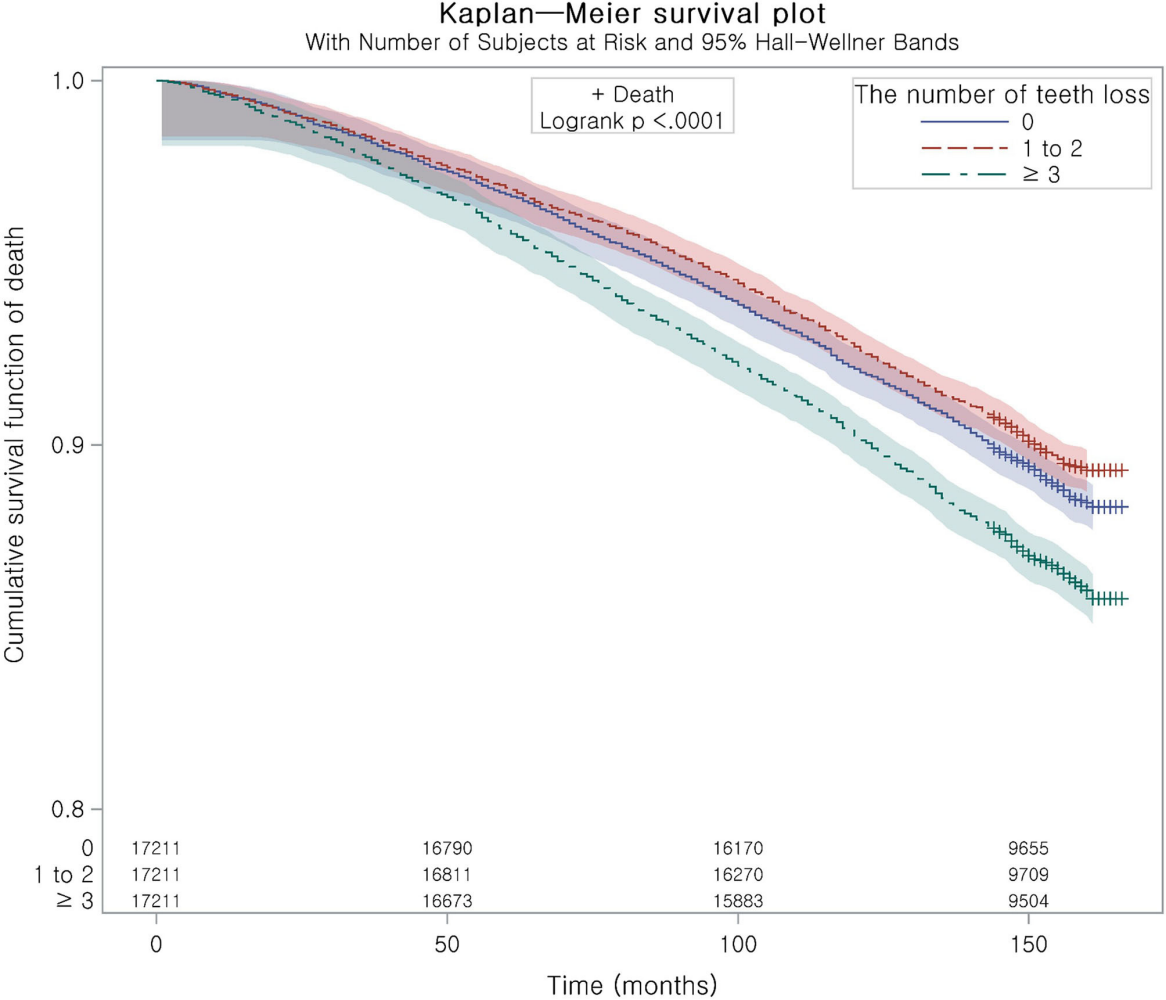
The Kaplan–Meier curve of mortality according to the number of missing teeth.

The incidence rate of all-cause mortality was 11.37, 8.48, and 9.23 per 1,000 person-year in the number of missing teeth ≥ 3, 1–2, and no missing teeth group (Table 2). The adjusted HR of mortality was 1.03 (95% CI = 0.96–1.10, *P* = 0.428) for the group with 1–2 missing teeth and 1.19 (95% CI = 1.12–1.27, *P* < 0.001) for the group with ≥ 3 missing teeth (Table 2). This was consistent with the Kaplan–Meier curve (Figure 3). Other primary outcomes of death are due to all-malignant neoplasm of digestive organs, all-malignant neoplasm of respiratory and intrathoracic organs, all-cerebrovascular diseases, and all-ischemic heart diseases, and secondary outcomes did not show an association with the number of tooth losses. The E-values of all-cause mortality were 1.21 for the group with 1–2 missing teeth and 1.67 for the group with ≥ 3 missing teeth (Supplementary Table 3).

**Table 2:** Association between the number of missing teeth, All-cause mortality, mortality of major diseases of death cause, and event of major diseases of death cause.

	**IR per 1,000 person-year**			**HRs in the 1–2 missing teeth group based** **on no missing teeth group**			**HRs in the ≥3 missing teeth group based** **on no missing teeth group**		
**Primary outcome**	**≥3 Missing teeth**	**1–2 Missing** **teeth**	**No missing teeth**	**IRD per 1,000** **person-years** **(95% CI)**	**Adjusted HRs** **(95% CI)^†^**	***p-*value**	**IRD per 1,000** **person-years** **(95% CI)**	**Adjusted HRs** **(95% CI)^†^**	***p*-value**
All-cause mortality (*n* = 5,904)	11.37	8.48	9.23	−0.75 (−1.32**–** −0.17)	1.03 (0.96**–**1.10)	0.435	2.14 (1.52**–**2.77)	1.19 (1.12**–**1.27)	<0.001^*^
All-Malignant neoplasms of digestive organs death (*n* = 1,258)	2.35	1.92	1.93	−0.02 (−0.29**–**0.25)	1.15 (1.10**–**1.33)	0.056	0.46 (0.18**–**0.75)	1.14 (0.99**–**1.30)	0.062
All-Malignant neoplasms of respiratory and intrathoracic organs death (*n* = 661)	1.24	0.93	1.08	−0.15 (−0.35**–**0.04)	1.01 (0.82**–**1.23)	0.946	0.15 (−0.06**–**0.36)	1.05 (0.87**–**1.26)	0.610
All-Cerebrovascular diseases death (*n* = 553)	1.04	0.78	0.93	−0.17 (−0.35**–**0.01)	0.93 (0.75**–**1.16)	0.517	0.11 (−0.08**–**0.30)	1.12 (0.92**–**1.37)	0.240
All-Ischemic heart diseases death (*n* = 302)	0.57	0.48	0.49	0.00 (−0.14**–**0.13)	1.09 (0.82**–**1.46)	0.546	0.03 (−0.11**–**0.17)	1.08 (0.82**–**1.43)	0.569
Secondary outcome									
Malignant neoplasms of digestive organs (*n* = 3,576)	6.07	6.16	5.82	0.34 (−0.14**–**0.82)	1.12 (1.04**–**1.22)	0.005	0.26 (−0.22**–**0.74)	0.99 (0.91**–**1.07)	0.795
Malignant neoplasms of respiratory and intrathoracic organs (*n* = 1,131)	1.93	1.68	1.99	−0.31 (−0.57**–** −0.05)	0.95 (0.82**–**1.10)	0.526	−0.06 (−0.33**–**0.21)	0.88 (0.76**–**1.01)	0.076
Cerebrovascular diseases (*n* = 8,419)	15.33	14.8	15.04	−0.23 (−1.02**–** 0.55)	0.99 (0.94**–**1.05)	0.754	0.28 (−0.51**–**1.08)	1.02 (0.97**–**1.08)	0.438
Ischemic heart diseases (*n* = 8,115)	14.08	15.6	14.32	1.27 (0.48**–**2.06)	1.05 (0.99**–**1.11)	0.088	−0.24 (−1.01**–**0.53)	0.97 (0.92**–**1.03)	0.325

*CI, confidence interval; HRs, hazard ratios; IR, incidence rate; IRD, incidence rate difference.*

* *Cox proportional hazard regression model, Significance at P < 0.05.*

† *The model was adjusted for age, sex, income, region, obesity, smoking, alcohol consumption, systolic blood pressure, fasting blood glucose, total cholesterol and CCI scores*.

In the subgroup analyses, the adjusted HR for the group with ≥ 3 missing teeth revealed statistical significance for each of the following subgroups: age of ≥ 55, BMI of < 23, BMI of ≥ 23, and high BP (each *P* < 0.05, Table 3). The interaction analyses demonstrated a significant interaction between the number of missing teeth and age, BMI, and SBP. On the other hand, the adjusted HR for the group with up to 2 teeth lost did not reach statistical significance in any subgroup.

**Table 3:** Association between the number of missing teeth with all-cause mortality in subgroups of age, obesity, and SBP.

**Characteristics**	**No. of death/ No. of participants**			**HRs in the 1–2 missing teeth group based** **on no missing teeth group**			**HRs in the** **≥3 missing teeth group based** **on no missing teeth group**			***P*-value of interaction**
	**≥3 Missing teeth**	**1–2 Missing teeth**	**No missing teeth**	**IRD per 1,000** **person-years** **(95% CI)**	**Adjusted HRs** **(95% CI)†**	**p-value**	**IRD per 1,000** **(95% CI)^†^** **person-** **years** **(95%** **CI)**	**Adjusted HRs** **(95% CI)^†^**	***p*-Value**	
Age < 55	417/8,334	330/8,334	292/8,334	0.38 (−0.10–0.86)	1.10 (0.93–1.28)	0.256	1.25 (0.74–1.77)	1.15 (0.99–1.34)	0.071	<0.001^*^
Age ≥ 55	1,870/8,877	1,406/8,877	1,589/8,877	−1.94 (−2.99– −0.89)	1.01 (0.94–1.09)	0.730	3.12 (1.98–4.27)	1.20 (1.12–1.28)	<0.001^*^	
BMI < 23	1,272/6,980	747/6,025	955/6,658	−1.77 (−2.86–0.52)	0.98 (0.89–1.08)	0.688	3.72 (2.54–6.05)	1.24 (1.14–1.35)	<0.001^*^	0.033^*^
BMI ≥ 23	1,015/1,0231	989/1,1186	926/1,0553	0.06 (−0.60–1.34)	1.05 (0.96–1.15)	0.313	1.00 (0.31–2.42)	1.13 (1.03–1.24)	0.007^*^	
SBP < 140 mmHg	1,223/1,0694	885/1,0584	1,004/1,1020	−0.65 (−1.31–0.69)	1.04 (0.96–1.14)	0.342	2.07 (1.36–3.54)	1.30 (1.20–1.41)	<0.001^*^	0.014^*^
SBP ≥ 140 mmHg	1,064/6,517	851/6,627	877/6,191	−1.21 (−2.30–0.89)	1.00 (0.90–1.11)	0.982	2.02 (0.85–4.32)	1.07 (0.97–1.18)	0.159	

*HRs, hazard ratios; IRD, incidence rate difference; SBP, systolic blood pressure.*

* *Cox proportional hazard regression model, Significance at P < 0.05.*

† *The model was adjusted for age, sex, income, region, obesity, smoking, alcohol consumption, systolic blood pressure, fasting blood glucose, total cholesterol and CCI scores*.

We displayed the mortality rate by each cause of death (Table 4 and Supplementary Table 4). The subdistribution HRs (SHRs) for mental disease and digestive disease were > 1 in the group with up to 2 teeth lost (Table 4). The SHRs for metabolic disease, digestive disease, trauma, and other diseases were > 1 in the group with ≥ 3 missing teeth. More detailed causes of death are described in Supplementary Table 2.

**Table 4:** Fine and Gray regression analysis in mortality between tooth loss 1 to 2 and tooth loss 0 groups and between tooth loss ≥ 3 and tooth loss 0 groups according to individual cause of death.

	**IR per 1,000 person-year**			**SHRs in tooth loss up to 2 group based on** **no tooth loss group**			**SHRs in tooth loss above 2 group based on** **no tooth loss group**		
**Cause of death**	**Tooth loss above** **2 group**	**Tooth loss up to** **2 group**	**No tooth loss** **group**	**IRD per 1,000** **person-years (95% CI)**	**Adjusted SHRs** **(95% CI)^†^**	***p*-Value**	**IRD per 1,000** **person-years** **(95% CI)**	**Adjusted SHRs** **(95% CI)^†^**	***p*-Value**
Infection	0.27	0.22	0.22	0.00 (−0.09–0.09)	1.09 (0.72–1.67)	0.675	0.05 (−0.04–0.15)	1.21 (0.81–1.80)	0.349
Neoplasm	4.26	3.54	3.74	−0.20 (−0.57–0.17)	1.08 (0.95–1.21)	0.245	0.52 (0.13–0.91)	1.08 (0.95–1.24)	0.242
Metabolic disease	0.47	0.31	0.29	0.02 (−0.09–0.13)	1.09 (0.75–1.58)	0.661	0.18 (0.06–0.30)	1.50 (1.07–2.09)	0.018^*^
Mental disease	0.11	0.11	0.07	0.05 (−0.01–0.10)	2.10 (1.03–4.25)	0.040^*^	0.04 (−0.01–0.10)	1.66 (0.85–3.24)	0.140
Neurologic disease	0.24	0.17	0.23	−0.05 (−0.14–0.04)	0.82 (0.52–1.30)	0.399	0.02 (−0.08–0.11)	1.15 (0.76–1.73)	0.509
Circulatory disease	2.03	1.64	1.84	−0.21 (−0.47–0.05)	0.98 (0.84–1.14)	0.742	0.18 (−0.09–0.45)	1.10 (0.96–1.27)	0.170
Respiratory disease	0.85	0.50	0.71	−0.21 (−0.36–−0.06)	0.90 (0.70–1.17)	0.440	0.14 (−0.03–0.32)	1.22 (0.97–1.52)	0.084
Digestive disease	0.51	0.40	0.29	0.11 (−0.01–0.22)	1.46 (1.03–2.05)	0.032^*^	0.22 (0.10–0.34)	1.53 (1.11–2.12)	0.010^*^
Muscular disease	0.07	0.05	0.04	0.02 (−0.03–0.06)	1.83 (0.69–4.84)	0.222	0.03 (−0.02–0.07)	1.91 (0.80–4.58)	0.147
Genitourinary disease	0.15	0.11	0.14	−0.03 (−0.10–0.04)	0.78 (0.43–1.40)	0.400	0.01 (−0.06–0.08)	1.07 (0.63–1.81)	0.799
Trauma	1.37	1.11	1.02	0.09 (−0.11–0.29)	1.20 (0.99–1.46)	0.062	0.35 (0.14–0.56)	1.33 (1.11–1.60)	0.002^*^
Others	0.83	0.46	0.64	−0.18 (−0.33–−0.04)	0.86 (0.66–1.13)	0.282	0.19 (0.03–0.36)	1.32 (1.05–1.65)	0.019^*^

*SHR, Subdistribution Hazard Ratio; IRD, incidence rate difference.*

* *Cox proportional hazard regression model, Significance at P < 0.05.*

† *The model was adjusted for age, sex, income, region, obesity, smoking, alcohol consumption, systolic blood pressure, fasting blood glucose, total cholesterol and CCI scores*.

## Discussion

Having ≥ 3 missing teeth was connected with greater mortality in the adult population. The positive connection between tooth loss and the mortality rate was maintained in most subgroups according to socioeconomic and comorbid conditions. According to the cause of death, the number of missing teeth was connected with elevated mortality rates due to neoplasms, metabolic disease, circulatory disease, respiratory disease, digestive disease, and trauma. This study improved previous studies on the mortality rate and tooth loss by extensively considering covariates, including past medical histories, socioeconomic factors, and lifestyle factors, which could be connected with both the number of missing teeth and mortality. In addition, the cause of mortality was classified and analyzed for its relationship with the number of missing teeth.

The increased risk of mortality associated with the number of missing teeth has been reported in a few previous studies (10, 22, 23). Adults with < 20 teeth demonstrated a 1.60 times higher risk of all-cause mortality than adults with ≥ 20 teeth in Japan (95% CI = 1.01–2.56, *P* = 0.047) (10). The number of missing teeth showed a dose-dependent influence on the risk of mortality in the elderly population (22, 23). Having ≥ 20 teeth, 10–19 teeth, and 1–9 teeth was associated with a 1.14 (95% CI = 1.06–1.23), 1·23 (95% CI = 1.15–1.31), and 1.35 (95% CI = 1.26–1.44) times higher risk of mortality, respectively (22). In addition to the considerable number of missing teeth, for instance, < 20 of remaining teeth, the present study demonstrated that missing 3 or more teeth could also contribute to the risk of mortality. Both the loss of the physiological functions of teeth and indirect effects on hygiene and inflammation could mediate the connection between tooth loss and mortality.

The discomfort of oral intake could induce a state of malnutrition, which may result in increased susceptibility to disease and mortality in patients with missing teeth. The missing teeth have been suggested to impair dietary intake, which in turn results in poor nutrition and weight loss (24). In patients with head and neck cancer, missing teeth were associated with 3.88 times (95% CI = 1.63–9.26) higher odds for poor dietary intake and 10.1 times (95% CI = 2.0–50) higher odds for weight loss of 5% or more (24). Nutritional impact symptoms, including chewing difficulty, dysphagia, loss of appetite, and feeling full, were also higher in the patients with missing teeth (OR = 1.11, 95% CI = 1.03–1.20) (24). In an aged population, the number of missing teeth was connected with 3.2 times higher odds of eating problems in an English observation study (25). Specifically, the maximum bite force and masticatory ability were lower in elderly patients with fewer than 20 teeth in Japan (26).

Moreover, the poor hygiene of patients with missing teeth could contribute to the increased risk of mortality. The major cause of missing teeth is periodontal disease. Because periodontal disease and dental caries accompany bacterial colonization and systemic inflammatory responses, systemic inflammation could mediate the increased risk of mortality (27). In addition, missing teeth can reduce self-esteem and quality of life, which may cause poor hygiene. Patients with missing teeth were reported to have a lower socioeconomic status (28, 29). In a 50-year or older Korean population, missing teeth were associated with 3.61%, 4.76, and 2.17% higher inequalities in aspects of parental education, personal education, and income, respectively (30). The low socioeconomic status of patients with missing teeth could result in poor hygiene, which can cause patients to be susceptible to morbidity and mortality.

According to the cause of death, mortality due to neoplasms and metabolic, circulatory, respiratory, and digestive diseases was positively associated with missing teeth. The missing teeth were reported to be connected with an increased risk of cardiovascular diseases (31). A meta-analysis demonstrated that the pooled risk ratio for the incidence of cardiovascular disease was 1.69–2.93 for patients with missing teeth (31). In addition, the main etiologies of tooth loss, dental caries, and periodontitis have been suggested to be connected with metabolic diseases, such as diabetes and metabolic syndrome (32, 33). The missing teeth and dental caries could impair nutritional intake and metabolic imbalance, which could induce metabolic diseases. On the other hand, metabolic diseases can increase susceptibility to dental caries and missing teeth due to dysregulated inflammation and immune functions. In patients with missing teeth, systemic inflammation and immune dysfunctions can also increase susceptibility to cancer, respiratory diseases, such as asthma and chronic obstructive pulmonary disease, and digestive diseases, such as liver diseases (34–36).

The positive association of ≥3 missing teeth and the risk of mortality was valid in many subgroups, including those of age ≥55, both sexes, various socioeconomic statuses, lifestyle factors, and comorbid conditions. Previous studies have yielded controversial associations of tooth loss with mortality according to age and sex. Although several prior studies have reported a risk of mortality related to missing teeth in the elderly population, a prospective study reported an association of missing teeth with mortality in a middle-aged population (40–64 years) but not in an elderly population (65–79 years) (12). According to sex, a prospective follow-up study in an 80-year-old population reported a stronger relationship between missing teeth with mortality in women (37). In contrast, another follow-up study in an 80-year-old population reported the connection of missing teeth with mortality in men but not in women (11). A smaller study population with limited age ranges may weaken the statistical power of previous studies. Because the present cohort harbored a large study population with a wider range of age groups, both men and women and many subgroups showed a relationship between missing teeth with the risk of mortality.

This study used large, nationwide, population cohort data. The groups with no missing teeth, 1–2 missing teeth, and ≥ 3 missing teeth were matched for age and sex. Several participants were excluded during the matching process. Thus, selection bias could be a concern in the present study. However, because age is a major determinant for both missing teeth and mortality, we matched this variable to minimize its potential confounding effects. In addition, most of the participants excluded during the matching process were in the no missing teeth group. The no missing teeth group was a control group in this study and matched with the study groups using random selection. Many variables were adjusted to minimize confounding effects, and subgroup analyses were conducted. The number of missing teeth was examined by dentists.

However, although the tooth exam was conducted by dentists, the etiology of missing teeth was not specified in this study. In this study, only 6.84% (3,531) of the participants were estimated to have fewer than 20 teeth. Thus, we categorized the groups according to the number of missing teeth: 0, up to 2, and more than 2 missing teeth. The possible impacts of underlying comorbidities on the occurrence of missing teeth could not be excluded in the present study. For instance, missing teeth can occur due to malnutrition related to pre-existing cancer and digestive diseases. Thus, although missing teeth are not the only etiology of mortality, this study has a clinical implication that missing teeth can be a predictive indicator for a greater risk of mortality. Although the HR of the present study was 1.19, the E-value was estimated to be as high as 1.67. In addition, other dental conditions, such as periodontal diseases and wearing dentures, were not included. The presence of the appropriate replacement of teeth could not be examined in the current cohort data. Previous studies reported that denture use was associated with a lower risk of mortality (22, 38). Although lifestyle factors and BMI were included as adjusted variables, the laboratory data were limited. For instance, the low-density lipoprotein cholesterol level was not available in the current study. Other residual confounders, such as educational status, dietary habits, and nutritional status, were not available in the current cohort data. Because dietary habits and nutritional states may impact both missing teeth and the risk of mortality, the association of missing teeth with the risk of mortality can mediate these unknown variables. Lastly, there were some missing values that were substituted with the average value of the total study population.

## Conclusion

The presence of at least three missing teeth was associated with a higher mortality rate in the adult population. According to the etiology of mortality, death from mental disease, digestive disease, and trauma was higher in the participants with missing teeth. Although many variables were adjusted in the present study, residual confounders could influence the association of the number of missing teeth with the risk of mortality.

## Data Availability Statement

The data analyzed in this study was obtained from The Korean National Health Insurance Service-Health Screening Cohort, which has restricted access. Requests to access these datasets should be directed to the Korean National Health Insurance Sharing Service.

## Ethics Statement

The studies involving human participants were reviewed and approved by the Ethics Committee of Hallym University (2019 - 10 - 023). The Ethics Committee waived the requirement of written informed consent for participation.

## Author Contributions

HC designed the study and drew the figures. DY, CL, MK, and HC analyzed the data. SK, J-HK, S-HB, and HC drafted and revised the manuscript. All authors have read and agreed to the published version of the manuscript.

## Funding

This work was supported in part by a research grant (NRF-2021-R1C1C1004986) from the National Research Foundation (NRF) of Korea. The funders had no role in the design of the study and collection, analysis, and interpretation of data and in writing the manuscript.

## Conflict of Interest

The authors declare that the research was conducted in the absence of any commercial or financial relationships that could be construed as a potential conflict of interest.

## Publisher's Note

All claims expressed in this article are solely those of the authors and do not necessarily represent those of their affiliated organizations, or those of the publisher, the editors and the reviewers. Any product that may be evaluated in this article, or claim that may be made by its manufacturer, is not guaranteed or endorsed by the publisher.

## References

[1] Kassebaum NJ Bernabe E Dahiya M Bhandari B Murray CJ Marcenes W. Global burden of severe tooth loss: a systematic review and meta-analysis. J Dent Res. (2014) 93(7 suppl):20S−8. 10.1177/0022034514537828 24947899 PMC4293725

[2] Ferreira RC de Magalhaes CS Moreira AN. Tooth loss, denture wearing and associated factors among an elderly institutionalised Brazilian population. Gerodontology. (2008) 25:168–78. 10.1111/j.1741-2358.2008.00214.x 18282145

[3] Gerritsen AE Allen PF Witter DJ Bronkhorst EM Creugers NH. Tooth loss and oral health-related quality of life: a systematic review and meta-analysis. Health Qual Life Outcomes. (2010) 8:126. 10.1186/1477-7525-8-126 21050499 PMC2992503

[4] Yamamoto T Kondo K Misawa J Hirai H Nakade M Aida J. Dental status and incident falls among older Japanese: a prospective cohort study. BMJ Open. (2012) 2:e001262. 10.1136/bmjopen-2012-001262 22855628 PMC4400665

[5] Aida J Kondo K Hirai H Nakade M Yamamoto T Hanibuchi T. Association between dental status and incident disability in an older Japanese population. J Am Geriatr Soc. (2012) 60:338–43. 10.1111/j.1532-5415.2011.03791.x 22211817

[6] Alvarenga MOP Ferreira RO Magno MB Fagundes NCF Maia LC Lima RR. Masticatory dysfunction by extensive tooth loss as a risk factor for cognitive deficit: a systematic review and meta-analysis. Front Physiol. (2019) 10:832. 10.3389/fphys.2019.00832 31333490 PMC6618904

[7] Qi X Zhu Z Plassman BL Wu B. Dose-response meta-analysis on tooth loss with the risk of cognitive impairment and dementia. J Am Med Dir Assoc. (2021) 22:2039–45. 10.1016/j.jamda.2021.05.009 34579934 PMC8479246

[8] Kim JH Oh JK Wee JH Kim YH Byun SH Choi HG. Association between tooth loss and Alzheimer's disease in a nested case-control study based on a national health screening cohort. J Clin Med. (2021) 10:3763. 10.3390/jcm10173763 34501210 PMC8432055

[9] Yamamoto T Kondo K Hirai H Nakade M Aida J Hirata Y. Association between self-reported dental health status and onset of dementia: a 4-year prospective cohort study of older Japanese adults from the Aichi Gerontological Evaluation Study (AGES) Project. Psychosom Med. (2012) 74:241–8. 10.1097/PSY.0b013e318246dffb 22408130

[10] Ishikawa S Konta T Susa S Ishizawa K Togashi H Ueno Y. Association between presence of 20 or more natural teeth and all-cause, cancer-related, and cardiovascular disease-related mortality: Yamagata (Takahata) prospective observational study. BMC Oral Health. (2020) 20:353. 10.1186/s12903-020-01346-6 33267797 PMC7709387

[11] Morita I Nakagaki H Kato K Murakami T Tsuboi S Hayashizaki J. Relationship between survival rates and numbers of natural teeth in an elderly Japanese population. Gerodontology. (2006) 23:214–8. 10.1111/j.1741-2358.2006.00134.x 17105502

[12] Ando A Tanno K Ohsawa M Onoda T Sakata K Tanaka F. Associations of number of teeth with risks for all-cause mortality and cause-specific mortality in middle-aged and elderly men in the northern part of Japan: the Iwate-KENCO study. Community Dent Oral Epidemiol. (2014) 42:358–65. 10.1111/cdoe.12095 24476489

[13] Kim SY Min C Oh DJ Choi HG. Tobacco smoking and alcohol consumption are related to benign parotid tumor: a nested case-control study using a national health screening cohort. Clin Exp Otorhinolaryngol. (2019) 12:412–9. 10.21053/ceo.2018.01774 31079445 PMC6787483

[14] Kim SY Min C Yoo DM Chang J Lee HJ Park B. Hearing impairment increases economic inequality. Clin Exp Otorhinolaryngol. (2021) 14:278–86. 10.21053/ceo.2021.00325 33781058 PMC8373834

[15] Kim SY Min C Oh DJ Choi HG. Bidirectional association between GERD and asthma: two longitudinal follow-up studies using a national sample cohort. J Allergy Clin Immunol Pract. (2020) 8:1005–13.e9. 10.1016/j.jaip.2019.10.043 31733335

[16] Kim SY Oh DJ Park B Choi HG. Bell's palsy and obesity, alcohol consumption and smoking: a nested case-control study using a national health screening cohort. Sci Rep. (2020) 10:4248. 10.1038/s41598-020-61240-7 32144385 PMC7060281

[17] Quan H Li B Couris CM Fushimi K Graham P Hider P. Updating and validating the Charlson comorbidity index and score for risk adjustment in hospital discharge abstracts using data from 6 countries. Am J Epidemiol. (2011) 173:676–82. 10.1093/aje/kwq433 21330339

[18] Haneuse S VanderWeele TJ Arterburn D. Using the E-value to assess the potential effect of unmeasured confounding in observational studies. JAMA. (2019) 321:602–3. 10.1001/jama.2018.21554 30676631

[19] VanderWeele TJ Ding P. Sensitivity analysis in observational research: introducing the E-value. Ann Intern Med. (2017) 167:268–74. 10.7326/M16-2607 28693043

[20] Localio AR Stack CB Griswold ME. Sensitivity analysis for unmeasured confounding: E-values for observational studies. Ann Intern Med. (2017) 167:285–6. 10.7326/M17-1485 28693037

[21] VanderWeele PD. Sensitivity analysis without assumptions. Epidemiology. (2016) 27:368–77. 10.1097/EDE.0000000000000457 26841057 PMC4820664

[22] Yuan JQ Lv YB Kraus VB Gao X Yin ZX Chen HS. Number of natural teeth, denture use and mortality in Chinese elderly: a population-based prospective cohort study. BMC Oral Health. (2020) 20:100. 10.1186/s12903-020-01084-9 32276615 PMC7147045

[23] Hayasaka K Tomata Y Aida J Watanabe T Kakizaki M Tsuji I. Tooth loss and mortality in elderly Japanese adults: effect of oral care. J Am Geriatr Soc. (2013) 61:815–20. 10.1111/jgs.12225 23590405

[24] Kubrak C Farhangfar A Woynorowski M Jha N Preshing W Baracos V. Dentition, nutritional status and adequacy of dietary intake in treatment naive head and neck cancer patients. Heliyon. (2020) 6:e03617. 10.1016/j.heliyon.2020.e03617 32258472 PMC7114741

[25] Steele JG Ayatollahi SM Walls AW Murray JJ. Clinical factors related to reported satisfaction with oral function amongst dentate older adults in England. Community Dent Oral Epidemiol. (1997) 25:143–9. 10.1111/j.1600-0528.1997.tb00912.x 9181288

[26] Tatematsu M Mori T Kawaguchi T Takeuchi K Hattori M Morita I. Masticatory performance in 80-year-old individuals. Gerodontology. (2004) 21:112–9. 10.1111/j.1741-2358.2004.00018.x 15185992

[27] Carrizales-Sepulveda EF Ordaz-Farias A Vera-Pineda R Flores-Ramirez R. Periodontal disease, systemic inflammation and the risk of cardiovascular disease. Heart Lung Circ. (2018) 27:1327–34. 10.1016/j.hlc.2018.05.102 29903685

[28] Hach M Christensen LB Lange T Hvidtfeldt UA Danielsen B Diderichsen F. Social inequality in tooth loss, the mediating role of smoking and alcohol consumption. Community Dent Oral Epidemiol. (2019) 47:416–23. 10.1111/cdoe.12468 31111525

[29] Ferreira RC Senna MIB Rodrigues LG Campos FL Martins A Kawachi I. Education and income-based inequality in tooth loss among Brazilian adults: does the place you live make a difference? BMC Oral Health. (2020) 20:246. 10.1186/s12903-020-01238-9 32887590 PMC7650222

[30] Han DH Khang YH. Lifecourse socioeconomic position indicators and tooth loss in Korean adults. Community Dent Oral Epidemiol. (2017) 45:74–83. 10.1111/cdoe.12262 27726172

[31] Beukers N Su N Loos BG van der Heijden G. Lower number of teeth is related to higher risks for ACVD and death-systematic review and meta-analyses of survival data. Front Cardiovasc Med. (2021) 8:621626. 10.3389/fcvm.2021.621626 34026863 PMC8138430

[32] Genco RJ Graziani F Hasturk H. Effects of periodontal disease on glycemic control, complications, and incidence of diabetes mellitus. Periodontol. (2000) 2020:83:59–65. 10.1111/prd.12271 32385875

[33] Furuta M Liu A Shinagawa T Takeuchi K Takeshita T Shimazaki Y. Tooth loss and metabolic syndrome in middle-aged Japanese adults. J Clin Periodontol. (2016) 43:482–91. 10.1111/jcpe.12523 26847391

[34] Michaud DS Liu Y Meyer M Giovannucci E Joshipura K. Periodontal disease, tooth loss, and cancer risk in male health professionals: a prospective cohort study. Lancet Oncol. (2008) 9:550–8. 10.1016/S1470-2045(08)70106-2 18462995 PMC2601530

[35] Dwibedi N Wiener RC Findley PA Shen C Sambamoorthi U. Asthma, chronic obstructive pulmonary disease, tooth loss, and edentulism among adults in the United States: 2016 behavioral risk factor surveillance system survey. J Am Dent Assoc. (2020) 151:735–44.e1. 10.1016/j.adaj.2019.07.032 31732091 PMC7531763

[36] Chen Y Yang YC Zhu BL Wu CC Lin RF Zhang X. Association between periodontal disease, tooth loss and liver diseases risk. J Clin Periodontol. (2020) 47:1053–63. 10.1111/jcpe.13341 32621350

[37] Ansai T Takata Y Soh I Awano S Yoshida A Sonoki K. Relationship between tooth loss and mortality in 80-year-old Japanese community-dwelling subjects. BMC Public Health. (2010) 10:386. 10.1186/1471-2458-10-386 20594306 PMC2903522

[38] Kowar J Stenport V Nilsson M Jemt T. Mortality in edentulous patients: a registry-based cohort study in Sweden comparing 8463 patients treated with removable dentures or implant-supported dental prostheses. Int J Dent. (2021) 2021:9919732. 10.1155/2021/9919732 34373694 PMC8349274

