# Supplementary material for: Is the Number of Missing Teeth Associated With Mortality? A Longitudinal Study Using a National Health Screening Cohort
Source: Front Med (Lausanne). 2022 Jun 21;9:837743. doi: 10.3389/fmed.2022.837743 (PMC9253612; doi:10.3389/fmed.2022.837743)
Supplement: Supplementary file 1 [file Table_1.docx]

**Supplement table** S1 The number of participants by the number of missing teeth.

| The number of missing teeth | | N (%) |
| --- | --- | --- |
| Total participants | | 51,633 (100.0) |
|  | 0 | 17,211 (33.3) |
|  | 1 | 10,247 (19.8) |
|  | 2 | 6,964 (13.5) |
|  | 3 | 4,802 (9.3) |
|  | 4 | 3,886 (7.5) |
|  | 5 | 1,868 (3.6) |
|  | 6 | 1,431 (2.8) |
|  | 7 | 917 (1.8) |
|  | 8 | 776 (1.5) |
|  | 9 | 456 (0.9) |
|  | 10 | 396 (0.8) |
|  | 11 | 290 (0.6) |
|  | 12 | 316 (0.6) |
|  | 13 | 177 (0.3) |
|  | 14 | 232 (0.4) |
|  | 15 | 150 (0.3) |
|  | 16 | 149 (0.3) |
|  | 17 | 113 (0.2) |
|  | 18 | 126 (0.2) |
|  | 19 | 97 (0.2) |
|  | 20 | 111 (0.2) |
|  | 21 | 85 (0.2) |
|  | 22 | 95 (0.2) |
|  | 23 | 61 (0.1) |
|  | 24 | 120 (0.2) |
|  | 25 | 81 (0.2) |
|  | 26 | 70 (0.1) |
|  | 27 | 50 (0.1) |
|  | 28 | 296 (0.6) |
|  | 29 | 6 (0.0) |
|  | 30 | 10 (0.0) |
|  | 31 | 4 (0.0) |
|  | 32 | 40 (0.1) |
