# Supplementary material for: Is the Number of Missing Teeth Associated With Mortality? A Longitudinal Study Using a National Health Screening Cohort
Source: Front Med (Lausanne). 2022 Jun 21;9:837743. doi: 10.3389/fmed.2022.837743 (PMC9253612; doi:10.3389/fmed.2022.837743)
Supplement: Supplementary file 3 [file Table_3.docx]

**Supplement table S3** E-value of HRs in the ≥3 missing teeth and 1-2 missing teeth groups based on no missing teeth group.

| Dependent variable | 1-2 missing teeth | | ≥3 missing teeth | |
| --- | --- | --- | --- | --- |
|  | Hazard ratios(95% CI) | E-value (CI) | Hazard ratios(95% CI) | E-value (CI) |
| All-cause mortality | 1.03 (0.96-1.10) | 1.21 (1.00) | 1.19 (1.12-1.27) | 1.67 (1.49) |
| All-Malignant neoplasms of  digestive organs death | 1.15 (1.10-1.33) | 1.57 (1.43) | 1.14 (0.99-1.30) | 1.54 (1.00) |
| All-Malignant neoplasms of respiratory and intrathoracic organs death | 1.01 (0.82-1.23) | 1.11 (1.00) | 1.05 (0.87-1.26) | 1.28 (1.00) |
| All-Cerebrovascular diseases death | 0.93 (0.75-1.16) | 1.36 (1.00) | 1.12 (0.92-1.37) | 1.49 (1.00) |
| All-Ischemic heart diseases death | 1.09 (0.82-1.46) | 1.40 (1.00) | 1.08 (0.82-1.43) | 1.37 (1.00) |
| Malignant neoplasms of digestive organs | 1.12 (1.04-1.22) | 1.49 (1.24) | 0.99 (0.91-1.07) | 1.11 (1.00) |
| Malignant neoplasms of respiratory and intrathoracic organs | 0.95 (0.82-1.10) | 1.29 (1.00) | 0.88 (0.76-1.01) | 1.53 (1.00) |
| Cerebrovascular diseases | 0.99 (0.94-1.05) | 1.11 (1.00) | 1.02 (0.97-1.08) | 1.16 (1.00) |
| Ischemic heart diseases | 1.05 (0.99-1.11) | 1.28 (1.00) | 0.97 (0.92-1.03) | 1.21 (1.00) |
