# Supplementary material for: Is the Number of Missing Teeth Associated With Mortality? A Longitudinal Study Using a National Health Screening Cohort
Source: Front Med (Lausanne). 2022 Jun 21;9:837743. doi: 10.3389/fmed.2022.837743 (PMC9253612; doi:10.3389/fmed.2022.837743)
Supplement: Supplementary file 4 [file Table_4.docx]

**Supplement table S4** Crude model of Fine and Gray regression analysis in mortality between 1-2 missing teeth and no missing teeth groups and between ≥3 missing teeth and no missing teeth groups according to individual cause of death.

| Cause of death | Proportion of Cause of death (n,%) | | | SHRs in 1-2 missing teeth group based on no missing teeth | | | SHRs in ≥3 missing teeth group based on no missing teeth | | |
| --- | --- | --- | --- | --- | --- | --- | --- | --- | --- |
|  | ≥3 missing teeth | 1-2 missing teeth | No missing teeth | IRD per 1000  person-years  (95% CI) | Crude SHRs  (95% CI)† | p-value | IRD per 1000  person-years  (95% CI) | Crude SHRs  (95% CI)† | p-value |
| All-cause death | 1,881 (100.0) | 1,736 (100.0) | 1,881 (100.0) |  |  |  |  |  |  |
| Infection | 56 (2.5) | 44 (2.5) | 45 (2.4) | 0.00 (-0.09 to 0.09) | 0.91 (0.64-1.48) | 0.915 | 0.05 (-0.04 to 0.15) | 1.24 (0.84-1.84) | 0.275 |
| Neoplasm | 872 (38.1) | 712 (41.0) | 762 (40.5) | -0.20 (-0.57 to 0.17) | 0.93 (0.84-1.032) | 0.177 | 0.52 (0.13 to 0.91) | 1.15 (0.04-1.26) | 0.005* |
| Metabolic disease | 97 (4.2) | 62 (3.6) | 59 (3.1) | 0.02 (-0.09 to 0.13) | 1.05 (0.74-1.50) | 0.784 | 0.18 (0.06 to 0.30) | 1.65 (1.19-2.27) | 0.002* |
| Mental disease | 23 (1.0) | 23 (1.3) | 14 (0.7) | 0.05 (-0.01 to 0.10) | 1.64 (0.84-3.19) | 0.143 | 0.04 (-0.01 to 0.10) | 1.64 (0.84-3.19) | 0.144 |
| Neurologic disease | 50 (2.2) | 35 (2.0) | 46 (2.5) | -0.05 (-0.14 to 0.04) | 0.76 (0.49-1.18) | 0.223 | 0.02 (-0.08 to 0.11) | 1.07 (0.73-1.62) | 0.685 |
| Circulatory disease | 415 (18.2) | 329 (19.0) | 376 (20.0) | -0.21 (-0.47 to 0.05) | 0.874 (0.75-1.01) | 0.074 | 0.18 (-0.09 to 0.45) | 1.04 (0.96-1.27) | 0.164 |
| Respiratory disease | 175 (7.7) | 101 (5.8) | 145 (7.7) | -0.21 (-0.36 to -0.06) | 0.70 (0.54-0.90) | 0.005* | 0.14 (-0.03 to 0.32) | 1.21 (0.97-1.50) | 0.093 |
| Digestive disease | 105 (4.6) | 81 (4.7) | 60 (3.2) | 0.11 (-0.01 to 0.22) | 1.35 (1.97-1.87) | 0.008 | 0.22 (0.10 to 0.34) | 1.75 (1.27-2.40) | <0.001* |
| Muscular disease | 14 (0.6) | 11 (0.6) | 8 (0.4) | 0.02 (-0.03 to 0.06) | 1.38 (0.55-3.42) | 0.492 | 0.03 (-0.02 to 0.07) | 1.75 (0.73-4.17) | 0.207 |
| Genitourinary disease | 30 (1.3) | 22 (1.3) | 28 (1.5) | -0.03 (-0.10 to 0.04) | 0.78 (0.45-1.37) | 0.396 | 0.01 (-0.06 to 0.08) | 1.07 (0.64-1.79) | 0.797 |
| Trauma | 280 (12.2) | 223 (12.9) | 208 (11.1) | 0.09 (-0.11 to 0.29) | 1.07 (0.89-1.30) | 0.465 | 0.35 (0.14 to 0.56) | 1.35 (1.13-1.61) | 0.001* |
| Others | 170 (7.4) | 93 (5.4) | 130 (6.9) | -0.18 (-0.33 to -0.04) | 0.71 (0.55-0.93) | 0.013* | 0.19 (0.03 to 0.36) | 1.31 (1.04-1.64) | 0.020* |

Abbreviation: SHR, Subdistribution Hazard Ratio; IRD, incidence rate difference
